# Defluorination of Polytetrafluoroethylene Surface by Hydrogen Plasma

**DOI:** 10.3390/polym12122855

**Published:** 2020-11-29

**Authors:** Alenka Vesel, Dane Lojen, Rok Zaplotnik, Gregor Primc, Miran Mozetič, Jernej Ekar, Janez Kovač, Marija Gorjanc, Manja Kurečič, Karin Stana-Kleinschek

**Affiliations:** 1 Department of Surface Engineering, Jozef Stefan Institute, Jamova cesta 39, 1000 Ljubljana, Slovenia; dane.lojen@ijs.si (D.L.); rok.zaplotnik@ijs.si (R.Z.); gregor.primc@ijs.si (G.P.); miran.mozetic@ijs.si (M.M.); jernej.ekar@ijs.si (J.E.); janez.kovac@ijs.si (J.K.); 2 Jozef Stefan International Postgraduate School, Jamova cesta 39, 1000 Ljubljana, Slovenia; 3 Faculty of Natural Sciences and Engineering, University of Ljubljana, Aškerčeva cesta 12, 1000 Ljubljana, Slovenia; marija.gorjanc@ntf.uni-lj.si; 4 Faculty of Mechanical Engineering, University of Maribor, Smetanova 17, 2000 Maribor, Slovenia; manja.kurecic@um.si; 5 Institute of Chemistry and Technology of Biobased Systems, Graz University of Technology, Rechbauerstraße 12, 8010 Graz, Austria; karin.stanakleinschek@tugraz.at

**Keywords:** polytetrafluoroethylene, fluorine depletion, hydrogen plasma, VUV radiation, surface modification, hydrophilic

## Abstract

Defluorination of polytetrafluoroethylene (PTFE) surface film is a suitable technique for tailoring its surface properties. The influence of discharge parameters on the surface chemistry was investigated systematically using radio-frequency inductively coupled H_2_ plasma sustained in the E- and H-modes at various powers, pressures and treatment times. The surface finish was probed by X-ray photoelectron spectroscopy (XPS) and time-of-flight secondary ion mass spectrometry (ToF-SIMS). The measurements of water contact angles (WCA) showed increased wettability of the pristine PTFE; however, they did not reveal remarkable modification in the surface chemistry of the samples treated at various discharge parameters. By contrast, the combination of XPS and ToF-SIMS, however, revealed important differences in the surface chemistry between the E- and H-modes. A well-expressed minimum in the fluorine to carbon ratio F/C as low as 0.2 was observed at the treatment time as short as 1 s when plasma was in the H-mode. More gradual surface chemistry was observed when plasma was in the E-mode, and the minimal achievable F/C ratio was about 0.6. The results were explained by the synergistic effects of hydrogen atoms and vacuum ultraviolet radiation.

## 1. Introduction

Fluorinated polymers are used in various applications [1]. They are renowned for their chemical inertness and thermal stability [2]. The chemical inertness does not allow for reasonable adhesion of any coating deposited by numerous techniques [3,4]. Whatever material is deposited, the surface energy of the coating is much larger than the energy of the substrate; therefore, thin films tend to form 3D particles spontaneously rather than a uniform film. Coatings from liquid solutions also do not adhere well on the surface, especially when the liquid is polar, i.e., water. In order to improve the adhesion of coatings, methods for increasing the surface energy of fluorinated polymers have been invented [5,6,7,8,9,10]. Fluorinated polymers are usually treated with aggressive chemicals which cause modification of the surface chemistry. Such techniques were invented in the 1960s, and some are still used nowadays. Common techniques include the application of sodium naphthalide [11], tetraalkylammonium radical anion salt, alkali metal vapours and amalgams [12], and electrochemical methods [13,14]. All these techniques represent an ecological hazard; therefore, researchers have been working on alternative techniques. A straightforward solution is irradiation of fluorinated polymers with beams of photons, electrons, or ions [15,16]. The insulating properties of fluorinated polymers make the techniques employing charged particles rather difficult, however.

A medium that contains both electrons, positively charged ions, and photons is gaseous plasma. Gaseous plasma is a state of gas also consisting of neutral particles (thereafter: radicals), which are chemically extremely reactive. For example, diatomic molecules are partially dissociated upon plasma conditions, and the atoms typically interact with most of the polymers even at room temperature. The atoms may bind to the surface of a polymer, thus forming functional groups of different polarity than those presented in fluorinated polymers. For example, oxygen plasma of a high density of oxygen atoms will cause functionalization of most polymers with various oxygen-containing functional groups [17]. While such treatments with oxygen plasma perform well for most types of polymers, they fail in the case of polytetrafluoroethylene (PTFE) for one simple reason: the binding energy of fluorine to carbon atoms is much larger than those of oxygen; therefore, a simple substitution of fluorine atoms on the surface of PTFE with oxygen atoms is energetically unfavourable. Treatment of fluorinated polymers by oxygen plasma will cause gradual etching of the polymer material rather than surface activation, as shown recently by Primc et al. [18]. 

Because classical plasma techniques fail in the case of PTFE, researchers have invented a variety of alternative methods. As early as 1987, Clark et al. [14] used a high vacuum plasma reactor to treat PTFE samples with hydrogen plasma. A radiofrequency (RF) generator coupled in a capacity mode was used for plasma generation. The maximum power was 10 W, and the treatment times were up to approximately 250 s. Upon such conditions, the surface film of PTFE was depleted of fluorine, which was proven by X-ray photoelectron spectroscopy (XPS). The water contact angle (WCA) showed moderate hydrophilicity of the treated samples after prolonged treatment. The minimum WCA was 50°. The authors explained the fluorine depletion by the interaction between hydrogen atoms and PTFE, causing the formation of HF molecules that were pumped away from the system.

About a decade later, Badey et al. [19] performed similar experiments, except that they used a powerful microwave (MW) generator for sustaining a dense plasma in a narrow quartz tube. PTFE samples were placed downstream from the centre of the discharge. The best results were observed at large gas flows and moderate discharge powers. The F/C ratio dropped from an original 2.45 to about 0.8. The WCA of water dropped from 115° to approximately 85° and diiodomethane from 84° to approximately 50°. The surface modifications were also monitored by secondary ion mass spectrometry (SIMS), and the authors found numerous C_x_H_y_ peaks after accomplishing the remote plasma treatment. As by Clark et al. [14], Badey et al. [19] also explained the observed results by the interaction between the polymer surface and neutral hydrogen atoms, causing the formation of HF molecules. In the same year, Yamada et al. [20] also applied remote H_2_ plasma treatment for the modification of PTFE samples. The plasma source was inductively coupled RF discharge. As by Badey et al. [19], the authors used a quartz tube for the discharge chamber and placed samples in the afterglow region. The authors reported similar results as Badey at large discharge powers. They also performed XPS characterization and found similar functional groups as Badey. The same group also used remote hydrogen plasma treatment to study the adhesion of Cu film on PTFE samples [21]. They obtained the minimum WCA of approximately 75° at the treatment time of approximately 2 min. In another paper, Inagaki et al. [22], used pulsed plasma treatment. An capacitiveRF discharge in the power range between 75 and 100 W was applied at the pressure of 13 Pa. The results and WCAs were slightly larger than in the case of using continuous plasma treatment. The same applied to the F/C ratio. 

König et al. [23] used plasmas sustained in various gases for modification of a plasma-deposited fluorocarbon polymer with the structure close to PTFE. Plasma was sustained by a MW discharge in the electron cyclotron resonance (ECR) mode. The working pressure was as low as 0.2 Pa. The maximum discharge power was 800 W. Samples were additionally biased using a capacitively coupled RF generator. The F/C ratio as deduced from the XPS survey spectra was 1.9 for the untreated material and dropped to 0.72 for plasma-treated samples. Simultaneously, the oxygen to carbon ratio O/C increased from 0.02 to 0.09. Such a surface finish enabled a slight decrease of WCA from the original 106° down to about 86°. 

Tanaka et al. [24] investigated defluorination of PTFE by a combination of atmospheric pressure glow plasma treatment and a chemical transport method. Plasma was sustained in a mixture of He and H_2_. The lowest F/C ratio reported for such plasma treatment was 0.4. The addition of oxygen in the gas mixture caused a further drop of the F/C concentration and appearance of a few atomic % of oxygen. More recently, Hunke et al. [25] performed direct treatment of PTFE powders in a low-pressure hydrogen plasma. They used a MW discharge and obtained a moderate decrease in the F/C ratio. The authors adopted the explanation provided previously by Inagaki et al. [22].

The review of the early work can be summarized as follows: the defluorination of PTFE surface occurs upon treatment with hydrogen plasma or its flowing afterglow and is explained by the interaction of hydrogen atoms with pristine material causing the formation of a dangling bond on one C atom. The dangling bond is quickly occupied with another H atom, forming the CHF group. This group may decompose by desorption of the HF molecule, and the result is the formation of the CF=CF group. The abundance of H atoms causes the gradual transformation of the polymer surface. A typical treatment time needed for observing a rather low F/C ratio is of the order of a minute.

In the present paper, we disclose experiments with hydrogen plasma performed in the same reactor at different conditions. Gaseous plasma was created in different discharge modes; therefore, some plasma parameters depended enormously on discharge conditions.

## 2. Materials and Methods 

PTFE foils were purchased from Goodfellow Ltd. (Huntingdon, UK). The foils with a thickness of 0.5 mm were cut to pieces of 10 × 10 mm^2^ and cleaned with ethanol, followed by drying at ambient conditions. 

**Figure 1:**
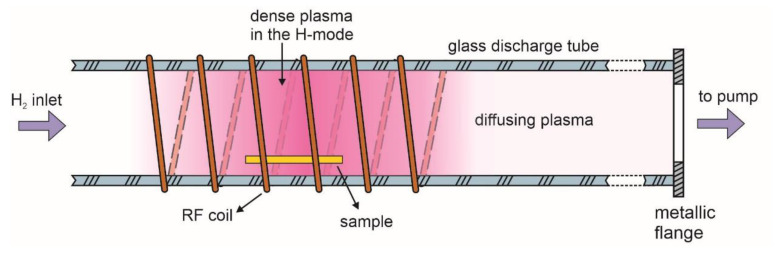
Schematic of the plasma set-up.

PTFE was treated in a glass discharge chamber using an electrodeless radio-frequency (RF) discharge. A schematic of the discharge chamber is shown in Figure 1. The discharge chamber was a long glass tube with a diameter of 4 cm. It was pumped on one side, and on the other side, H_2_ gas was introduced through the flow controller. The vacuum system was sealed with rubber gaskets. It was pumped with a two-stage rotary pump of a nominal pumping speed of 80 m^3^/h. The experimental conditions, therefore, enabled achieving the ultimate pressure just below 1 Pa after pumping for a reasonable time. Optical emission spectroscopy (OES) was used to check any gaseous impurities in the discharge chamber. Spectral features of any trace gases were below the detection limit of the spectrometer; therefore, the system was hermetically tight, and the residual atmosphere contained water vapour only. Plasma was sustained within the coil, as shown in Figure 1 and diffusing plasma expanded far away from the coil. The coil was connected to the RF generator via a matching network. The matching network allowed for coupling optimization to run the plasma mostly in the H-mode, depending on the power. The generator operated at the standard frequency of 13.56 MHz and adjustable output power up to 1000 W. The samples were treated at various conditions, i.e., various powers from 100 to 1000 W, hydrogen pressures from 10 to 60 Pa, and treatment times from 0.5 to 12 s.

The surface wettability of samples was measured using a Drop Shape Analyser DSA-100 (Krüss GmbH, Hamburg, Germany). A static contact angle was measured using a sessile drop method. The volume of a drop was 1 µL. MiliQ water was used for determination of the wettability.

Surface modifications were probed by XPS and time-of-flight secondary ion mass spectrometry (ToF-SIMS). The XPS characterization was performed using an XPS instrument (model TFA XPS from Physical Electronics, Münich, Germany). The samples were irradiated with monochromatic Al Kα_1,2_ radiation with the photon energy of 1486.6 eV. Spectra were measured at an electron take-off angle (TOA) of 45°. Selected samples were also measured at various TOAs to manipulate the detection depth of XPS. Survey spectra were acquired at a pass-energy of 187 eV using an energy step of 0.4 eV. High-resolution carbon C1s spectra were measured at a pass-energy of 23.5 eV using an energy step of 0.1 eV. An additional electron gun was used for compensation of the surface charge. Spectra were calibrated by adjusting the C1s peak corresponding to CF_2_ groups to 292 eV. The measured spectra were analyzed using MultiPak v8.1c software (Ulvac-Phi Inc., Kanagawa, Japan, 2006) from Physical Electronics, which was supplied with the spectrometer. Linear background subtraction was used. 

ToF-SIMS analyses were performed using a ToF-SIMS 5 instrument (ION-TOF, Münster, Germany) equipped with a bismuth liquid metal ion gun with a kinetic energy of 30 keV. The analyses were performed in an ultra-high vacuum of approximately 10^−7^ Pa. The ToF-SIMS spectra were measured by scanning a Bi^3+^ cluster ion beam over the surface spot of approximately 100 × 100 µm^2^. An electron gun was used to allow for charge compensation on the sample surfaces during the analysis. Positive and negative ion spectra were measured

## 3. Results and Discussion

### 3.1. Modification of the Surface Wettability

**Figure 2:**
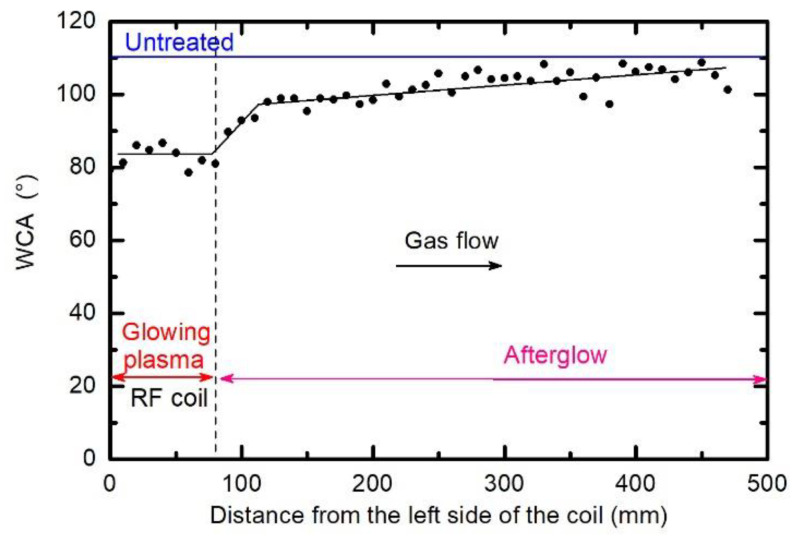
Variation of water contact angle (WCA) on the polytetrafluoroethylene (PTFE) surface of the samples arranged along the discharge tube. The treatment time was 1 s, the hydrogen pressure was 25 Pa, and the discharge power was 400 W.

Samples were placed at different positions along the discharge tube to measure the gradient in the surface wettability. Some of them were placed inside the RF coil, and many were placed away from the coil, in the direction of the pump duct. The water contact angle was measured for all these samples, and the result is presented in Figure 2. Plasma treatment time in all cases was 1 s, the hydrogen pressure was 25 Pa, and the discharge power was 400 W. The WCA for the untreated sample was approximately 110°. One can observe a gradual increase in the WCA with the increasing distance from the RF coil. The samples treated within the coil assume the WCA of approximately 83°, which is the value already reported by previously cited authors. This value is typical for oxygen-free polymers such as polyolefins. Such a rather low WCA extends a few cm away from the coil, which is explained by the simple fact that a dense plasma in the H-mode was not limited to the coil only, but also stretched outside of the coil as shown schematically in Figure 1. Away from the coil, the WCA increases to approximately 100° within a distance of several cm. Thereafter, the WCA increases rather linearly with increasing distance, and at large distances approaches the value typical for the untreated sample. The measured points scatter somehow, but the trend is obvious and is presented by straight lines. The results summarized in Figure 2, therefore, suggest almost complete defluorination of the PTFE samples upon treatment with a dense, glowing hydrogen plasma, and a more gradual activation upon treatment with a diffusing plasma, which in our case is a result of a weak capacitive coupling between the coil and the metallic pump duct.

**Figure 3:**
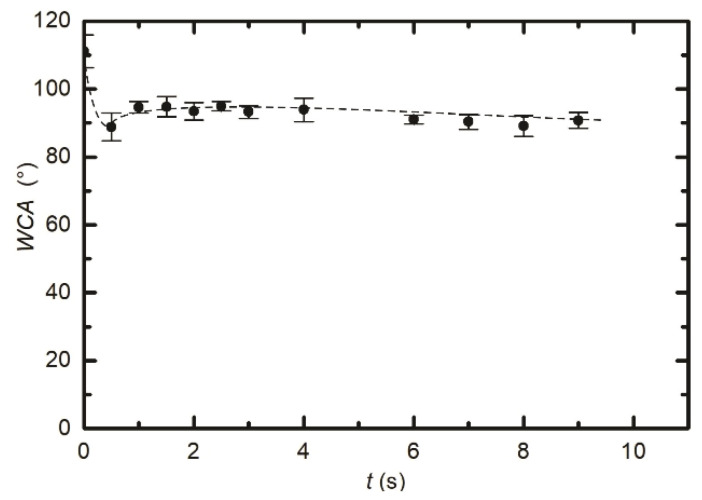
Variation of surface wettability with treatment time. The samples were inside the radiofrequency (RF) coil. Discharge power and hydrogen pressure were constant at 400 W and 25 Pa, respectively.

**Figure 4:**
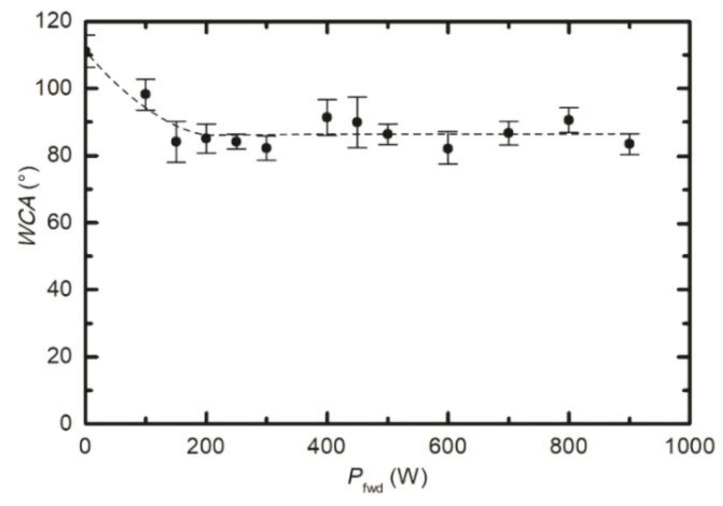
Variation of surface wettability with the forward discharge power. Treatment time and hydrogen pressure were constant at 1 s and 25 Pa, respectively.

**Figure 5:**
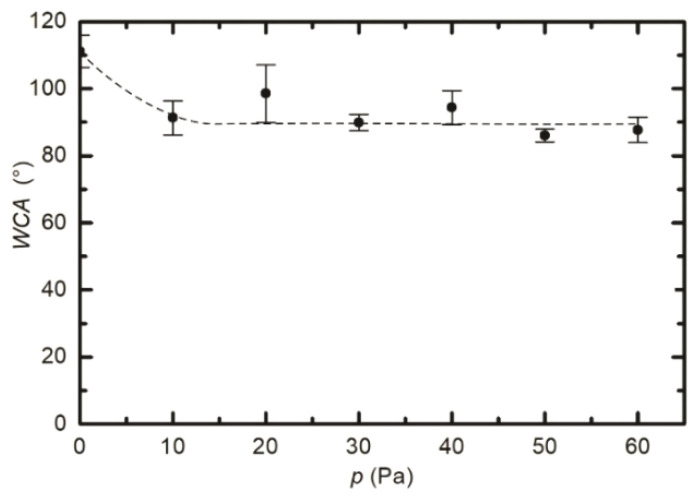
Variation of surface wettability with hydrogen pressure. Treatment time and discharge power were constant at 1 s and 400 W, respectively.

As already reported by numerous authors, the WCA depends on numerous treatment parameters, including the treatment time, the pressure, and the power absorbed by the gaseous plasma [20,21,22,26,27]. To get additional information about the evolution of the surface wettability, we treated several samples inside the RF coil at various conditions. Figure 3 represents the WCA versus plasma treatment time. We adopted the same parameters as in Figure 2, i.e., the pressure of 25 Pa and the power of 400 W. It seems that the WCA in Figure 3 does not really depend on treatment time at these particular discharge conditions. All values lay at approximately 90°, with the exception of the first measurement corresponding to the lowest treatment time of 0.5 s where the standard deviation is rather large. The almost constant WCA, as revealed from Figure 3, is explained by the saturated defluorination of the surface. This effect will be further discussed later. Figure 4 is a plot of the WCA versus the discharge power for the treatment time of 1 s and the pressure of 25 Pa. Again, one can observe a rather constant WCA, except for the measurement performed at the lowest power of 100 W. The WCA also does not depend much on the H_2_ pressure in the discharge tube, as shown in Figure 5. Figure 3, Figure 4 and Figure 5, therefore indicate that the surface activation is accomplished within the second of plasma treatment providing the discharge power is reasonably large, and the pressure is in the range between 10 and 60 Pa.

The water contact angles, as observed from Figure 2, Figure 3, Figure 4 and Figure 5, indicate a rather marginal increase of the PTFE wettability, i.e., the WCA remained above 80°. Such a WCA was reported already by Badey at al. [4] and Konig at al. [8]. Somehow lower WCA of 75° was reported by Yamada et al. [5], and the WCA of about 50° was found by Clark et al. [2]. The discrepancy was explained recently by Primc [3] who showed that the WCA on the surface of fluorinated polymers depends on the concentration of other elements. Even a small concentration of oxygen, for example, caused a decrease of the WCA below the values typical for polyolefins (about 80°). 

The results of the surface wettability as probed by the WCA do not show any obvious trend. Because this technique does not reveal the chemical modifications taking place on the sample surface upon plasma treatment, we additionally performed research on the composition and structure of the surface film as probed by XPS and ToF-SIMS to further elaborate details about the surface chemistry.

### 3.2. Chemical Modifications as Determined by X-ray Photoelectron Spectroscopy (XPS)

**Figure 6:**
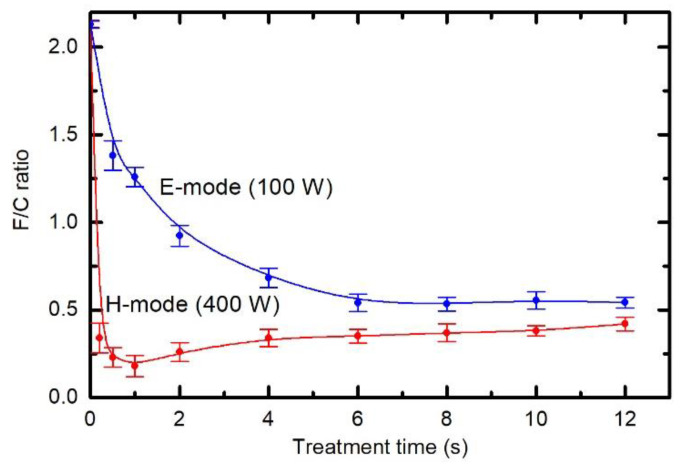
Variation of the X-ray photoelectron spectroscopy (XPS) F/C ratio versus treatment time for two different powers. Hydrogen pressure was constant at 25 Pa.

Figure 6 shows the XPS F/C ratio versus the treatment time when plasma was sustained at a large power of 400 W (lower curve) and at low power of 100 W (upper curve). See also supplementary Figures S1 and S2 showing individual survey spectra and elemental composition versus treatment time. The upper curve of Figure 6 corresponds to the same experimental conditions as the WCA measurement at the discharge power of 100 W in Figure 4, which reveals incomplete activation of the surface at 100 W after the treatment for 1 s. Examining Figure 6, it is obvious that incomplete surface activation is a consequence of the insufficient removal of fluorine from the surface of PTFE, because the F/C concentration after the treatment for 1 is still about 1.25, thus far from complete defluorination.

The upper curve in Figure 6 shows a gradual decrease of the F/C ratio with increasing treatment time. Gaseous plasma at the pressure of 25 Pa and RF power of 100 W is sustained in the E-mode. Because the matching network was optimized for coupling in the H-mode, a significant fraction of the RF power was reflected and thus not absorbed by the gaseous plasma. The power absorbed in plasma for the case of the upper curve of Figure 6 is, therefore, well below 100 W. Still, the surface film as probed by XPS is depleted of fluorine even after several seconds of plasma treatment and approaches a value of approximately 0.6. It seems that such a concentration of fluorine is about all one can achieve upon treatment of PTFE in hydrogen plasma sustained in the E-mode.

The mechanism is completely different when plasma is sustained in the H-mode (lower curve). In this case, the surface film is depleted of fluorine even after approximately 0.2 s of plasma treatment. The F/C ratio further decreases with increasing treatment time until a minimum at approximately 1 s appears. Thereafter, a gradual but slow increase in the F/C ratio is observed. After approximately 10 s, the F/C ratio assumes a value of approximately 0.4. This value is lower than what is achievable in the E-mode.

A huge difference in the surface composition between the E- and H-modes, as evident from Figure 6, should be explained by different mechanisms. Gaseous plasma in the H-mode is an extensive source of vacuum ultraviolet (VUV) radiation [28]. Recently, Fantz et al. investigated the details regarding the radiation arising from H_2_ plasma sustained by an inductively coupled RF discharge in the H-mode [29]. The discharge configuration was almost identical to the one shown in Figure 1. Fantz et al. showed that approximately 10% of the available discharge power is transformed into radiation in the VUV range. This radiation causes bond scission in the surface film of a thickness of the order of a penetration depth for VUV photons. The penetration depth depends on the wavelength (i.e., photon energy) but is definitely larger than the escape depth of photoelectrons. By considering this fact, one can assume a rather homogenous treatment of the surface film with the VUV. The bond scission enables further reactions, including the interaction with H atoms. The H atoms attack the dangling bonds as already reported by Badey et al. [19]. Furthermore, they interact with F atoms forming HF molecules, which are desorbed upon vacuum conditions and pumped away. The combination of bond scission caused by absorption of VUV radiation and chemical interaction with H atoms should, therefore, ensure an F-free surface. Such an effect cannot be confirmed from XPS measurements because of the final escape depth of photoelectrons. It will be shown later in this paper that the best technique to prove the F-free surface is ToF-SIMS. 

After prolonged treatment of PTFE samples in the H-mode, the F/C ratio does not remain constant but increases slowly with increasing treatment time. Such an increase may be explained by a different thickness of the F-depleted surface film rather than by incomplete defluorination. This effect will be explained later by using angular-resolved XPS characterization (AR-XPS).

**Figure 7:**
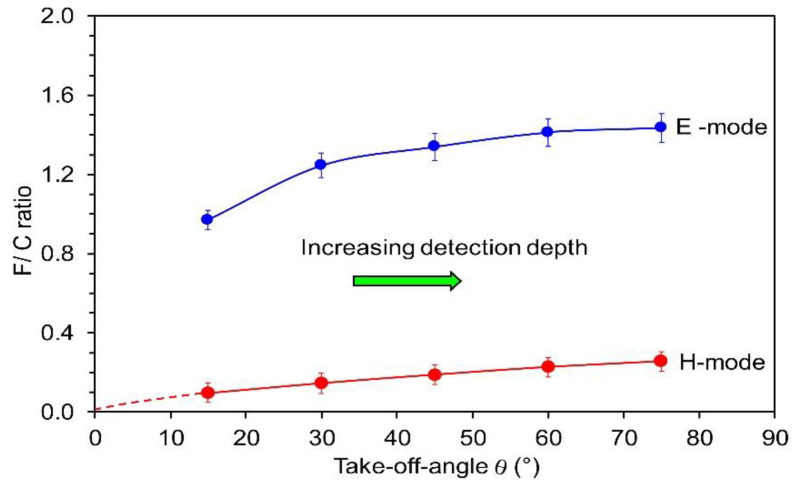
Variation of the AR-XPS F/C ratio versus the photoelectron take-off angle for two different powers and the same pressures of 25 Pa and treatment times of 1 s.

When plasma is in the E-mode, the minimum in the F/C ratio is not observed, which may be a consequence of the fact that such plasma is not a significant source of VUV radiation. The luminosity of plasma in the E-mode is typically 3–4 orders of magnitude lower than in the H-mode at this pressure, i.e., 25 Pa. The difference in plasma luminosities between the E- and H-modes normally increases with increasing pressure, as shown by Fantz et al. [29]. The F/C ratio, however, does not deviate for orders of magnitude but is comparable for long treatment times. The comparison of the two curves in Figure 6, therefore, indicate that the intensive VUV radiation only accelerates defluorination of the surface film. A rather low F/C ratio observed after the treatment in the E-mode should be because of other mechanisms. It was already mentioned that H atoms attack the polymer surface and dangling bonds, causing the formation of the volatile HF molecule. Badey et al. [19], as well as Yamada et al. [20], investigated the evolution of the F/C ratio in the flowing afterglow where VUV radiation is negligible, but the density of H atoms is still significant. They found moderate F/C ratios of approximately 0.8. Somehow, lower F/C ratio was also reported by König et al. [23]. Unlike VUV radiation, the H atoms do not penetrate into the solid polymer; therefore, the chemical modification should be limited to a thinner film in the E-mode as compared to the H-mode. Such a difference in the thickness of the well-affected film can explain the fact that the achievable F/C ratio in the E-mode is lower than in the H-mode. A confirmation for the statement about the F-free surface for the H-mode is provided in Figure 7, which shows the F/C ratios as deduced from AR-XPS. The lower curve is for the sample treated for 1 s in the H-mode, whereas the upper for the sample treated for the same time in the E-mode. The F/C ratio in the H-mode gradually increases with increasing take-off angle *θ* (TOA). Detection depth (*d*) is given by the following relation, d=3λ⋅sin(θ), where *λ* is the inelastic mean free path of photoelectrons. The detection depth thus increases with increasing TOA. The gradual increase in the F/C ratio is explained by the formation of the almost F-free surface as well as a subsurface layer with a thickness close to the detection depth of XPS. Extrapolation of the lower curve to the TOA *θ* = 0° reveals the F/C ratio is practically zero on the surface. Unfortunately, measurements at extremely low TOA are not feasible.

As shown before in Figure 6, a defluorination is incomplete when plasma is in the E-mode for 1 s. The upper curve in Figure 7 confirms this. However, it is interesting that the F/C ratio in Figure 7 increases by a factor of approximately 1.5 when the TOA is increased from *θ* = 15 to 75°. Obviously, the surface film contains much less fluorine than the subsurface one, and there is a significant gradient in F concentration. Any extrapolation of the curve towards the TOA *θ* = 0° would be speculation; therefore, it is not shown in Figure 7.

**Figure 8:**
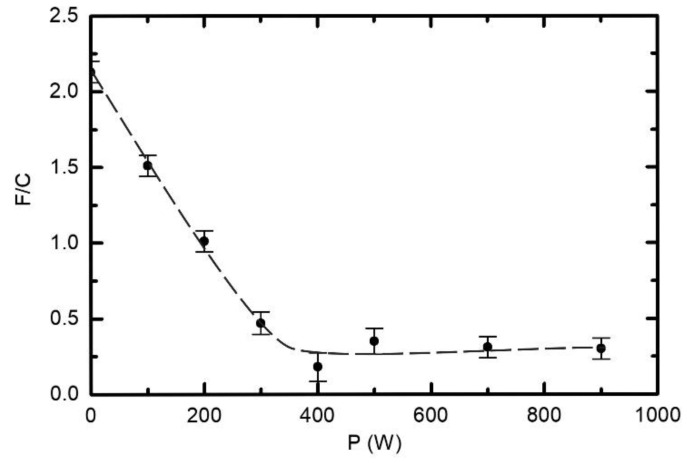
Variation of the F/C ratio versus the discharge power. Treatment time and H_2_ pressure were constant at 1 s and 25 Pa, respectively.

Figure 6 and Figure 7 indicate large differences in surface chemistry, depending on the type of discharge. The differences can be further elaborated by performing measurements at a fixed treatment time and pressure, but different RF powers. The result is plotted in Figure 8. Here, the F/C ratio reaches the minimum at 400 W. At larger powers, the F/C is somehow slightly larger. The behaviour of the curve for powers between 400 and 900 W is similar to the lower curve in Figure 6. In both cases, the fluence of VUV radiation increases with increasing value at the abscissa. More interesting is the behaviour at lower powers. One can observe a gradual and rather linear decrease of the F/C ratio with increasing power. The discharge power of 300 W results in the F/C ratio of approximately 0.5, similar to what is observed in Figure 6 for the upper curve after prolonged plasma treatment. Comparison of Figure 6 and Figure 8, therefore, indicates that the key parameter governing the surface chemistry is the fluence of reactive species and/or VUV radiation rather than discharge power or treatment times. Nonetheless, it is important that the surface finish in the E-mode as deduced from XPS is different to that in the H-mode. In addition to Figure 8, see also supplementary Figures S3 and S4 showing individual survey spectra and elemental composition versus the discharge power.

The variation of the F/C ratio versus the discharge power (Figure 8) is different to the behaviour of WCA (Figure 4). Comparison of Figure 4 and Figure 8 indicates that even an incomplete defluorination causes the drop of the water contact angle to values typical for polyolefins. 

**Figure 9:**
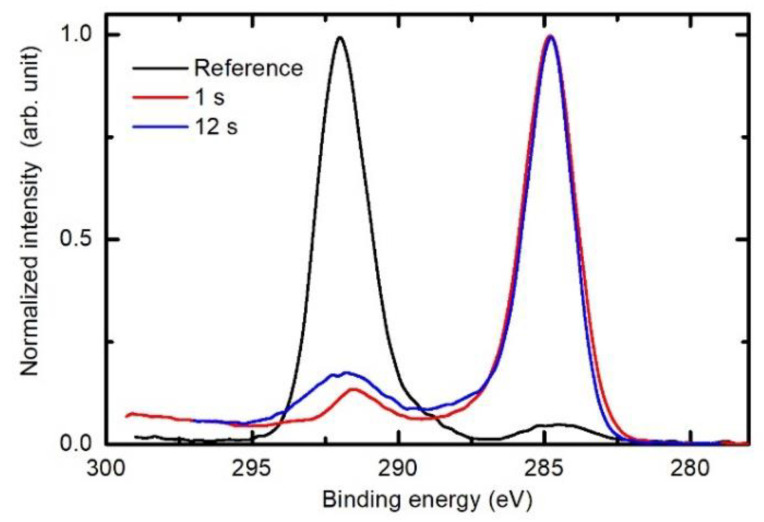
Comparison of XPS high-resolution C1s spectra of the untreated PTFE, and PTFE exposed to H_2_ plasma for 1 and 12 s. The discharge power and the pressure were constant at 400 W and 25 Pa, respectively.

Figure 9 reveals the high-resolution C1s spectra for the untreated sample and samples treated in the H-mode for 1 and 12 s. There is a huge difference between the untreated and treated samples. The untreated sample contains only carbon bonded in CF_2_ groups. The C1s peak, therefore, appears at a binding energy of approximately 292 eV. There is also a small peak at approximately 285 eV, which corresponds to surface impurities. The treated samples exhibit the opposite behaviour—the major peak is at about 285 eV, whereas some features also persist at higher binding energies up to approximately 294 eV. Taking into account Figure 7, and the discussion thereafter, it can be concluded that the features correspond to degraded PTFE-like film, whereas the main peak at 285 eV corresponds to F-depleted surface film. Therefore, we can conclude that the surface film of samples treated in H_2_ plasma in the H-mode contains olefin-like carbon. It is interesting that the intensity in the range of binding energies from about 287 and 294 eV is slightly larger for the case of 12 s than for 1s of plasma treatment. The reasons for this have already been elaborated when explaining the behaviour of the lower curve in Figure 6 and Figure 7.

**Figure 10:**
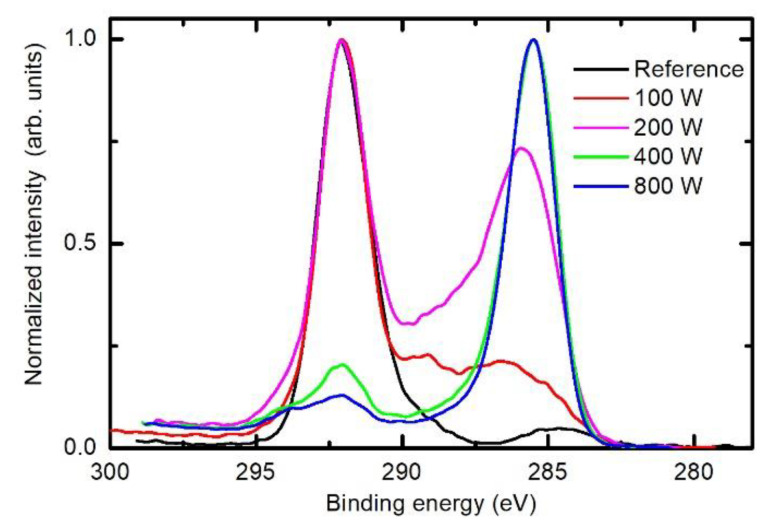
Comparison of the selected XPS high-resolution C1s spectra of the PTFE samples treated for various powers. The treatment time and the pressure were constant at 1 s and 25 Pa, respectively.

By considering the upper discussion, the C1s peak of samples treated in the E-mode should differ from those in the H-mode. Figure 10 represents solid proof of the evolution of the surface chemistry upon treatment of PTFE samples in the E- and H-modes. The behaviour of the two curves at 400 and 800 W was already explained. Interesting, and sound with the previous discussion, is the behaviour of the curves acquired after the treatment at discharge powers of 100 and 200 W. In both cases, plasma was in the E-mode. The curve at 100 W indicates that the rather intact PTFE still persists, but a fraction is already modified enough to form various carbon chemistries including intermediate ones, as already reported by Badey et al. [19]. A well-expressed peak is observed at approximately 292 eV, as well as a broad feature between 292 and 285 eV. This indicates that CH groups have already appeared on the surface, but the thin surface film also contains other groups with binding energies between 292 and 285 eV. The spectrum corresponding to treatment at 200 W shows a gradual deviation from the untreated PTFE to a surface film of polyolefin-like structure. As discussed above and in keeping with the observations in Figure 6 and Figure 7, the thickness of the F-free surface film in the E-mode is much lower than the escape depth of photoelectrons, so the significant contribution of photoelectrons with binding energies of 292 eV still persists. In between the two well-defined peaks at 292 and 285 eV, there is a rich structure, which could be deconvoluted almost arbitrarily taking into account a variety of functional groups as well as peculiarities of XPS regarding the interpretation of F-containing functional groups [30]. For this reason, we made no attempt to deconvolute C1s peaks shown in Figure 10.

### 3.3. Chemical Modifications as Determined by Time-of-Flight Secondary Ion Mass Spectrometry (ToF-SIMS)

**Figure 11:**
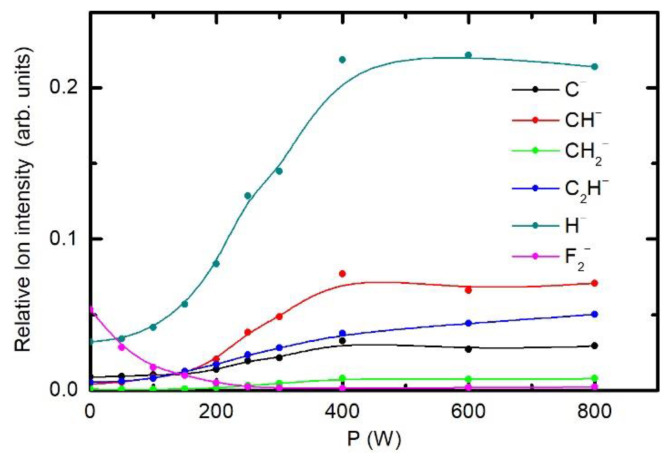
Variation of time-of-flight secondary ion mass spectrometry (ToF-SIMS) intensities of selected negative ions. The pressure was 25 Pa and treatment time 1 s.

**Figure 12:**
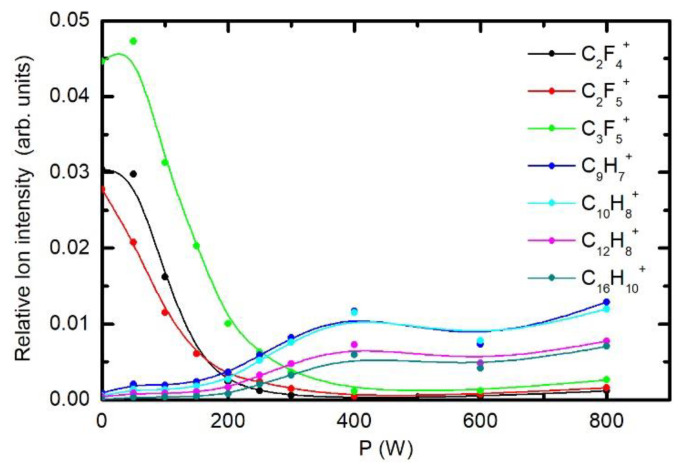
Variation of ToF-SIMS intensities of selected positive ions. The pressure was 25 Pa and treatment time 1 s.

Deconvolution of C1s XPS spectra of fluorinated samples definitely represents a scientific challenge. Therefore, it is useful to characterize selected samples also with an alternative technique, such as ToF-SIMS. The evolution of the ToF-SIMS spectral features was investigated systematically for the treatment time of 1 s and hydrogen pressure of 25 Pa. We varied the discharge power to obtain further insight into the surface chemistry. Some examples of the selected positive in negative ion spectra of the samples are shown in Supplementary Figures S5–S8. Figure 11 shows the selective negative ion intensities and Figure 12 positive ion intensities. One can observe a gradual decreasing of the F^2−^ intensity versus the discharge power. This behaviour indicates depletion of the surface film as probed by ToF-SIMS. It should be noted that the surface sensitivity of ToF-SIMS is superior in comparison to XPS. Simultaneous to decreasing of the F^2−^ intensity, a number of fluorine-free ions appear in the ToF-SIMS negative ion spectra. All of them keep increasing with increasing discharge power up to the power of 400 W. Thereafter, the intensity of fluorine-free ions in ToF-SIMS spectra remains constant. This observation is in keeping with results presented in Figure 11, where exactly the same effect was observed. Unfortunately, ToF-SIMS does not allow for reliable quantification of the measured spectra. The drop of the intensity of F^2−^ for more than a factor of 50 indicates practically F-free surface. 

Figure 12 shows the behaviour of positive ion fragments. The intensities of F-containing ions decreases monotonously with increasing discharge power, and the minimum is observed at the power of 400 W. By contrast, the ions containing hydrogen increase gradually up to the power of 400 W and remain fairly intact thereafter. The ToF-SIMS results are, therefore, in keeping with those obtained by XPS.

## 4. Conclusions

Systematic characterization of PTFE was performed to reveal the kinetics of fluorine depletion of the surface film of this polymer upon treatment with hydrogen plasma at various conditions. Unlike previous authors, we concentrated on rather short treatment times of the order of a second. Such short times are attractive for any application of gaseous plasma technology for surface processing of products made from fluorinated polymers. A broad range of parameters was found useful for the depletion of the surface film. Nominal discharge powers of the RF generator of as low as 100 W are capable of depletion of the surface film within several seconds of plasma treatment. The intensity of surface chemical reactions increases with increasing discharge power, and the reactions become almost instant once the discharge is in the H-mode. A significant difference in the surface finish between the discharge modes was observed. In the case the discharge is in the E-mode, the F concentration decreases monotonously with increasing treatment time, and the minimal achievable F/C ratio is just above 0.5. By contrast, when the plasma is in the H-mode, a well-defined minimum in the F concentration occurs at rather short treatment times. The minimal F/C ratio as deduced from the XPS spectra acquired at standard take-off angle is about 0.2. The surface layer of the polymer, however, is almost free from fluorine, which is proved by ARXPS as well as by the behaviour of specific ion fragments acquired by ToF-SIMS. Experiments performed at different discharge conditions qualitatively indicate that the key parameter governing the surface finish is the fluence of reactive plasma species. Unfortunately, our experimental set-up did not allow for reliable determination of the radiation in the VUV range. The surface finish versus the fluence of VUV radiation upon treatment of fluorinated polymers with hydrogen plasma, therefore, remains a scientific challenge.

## Supplementary Materials

The following are available online at https://www.mdpi.com/2073-4360/12/12/2855/s1, Figure S1: XPS survey spectra of PTFE treated in hydrogen plasma for various treatment times. Hydrogen pressure was constant at 25 Pa and the discharge power was: (a) 100 W and (b) 400 W., Figure S2: XPS surface composition of PTFE treated in hydrogen plasma at various treatment times: (a) for discharge power of 100 W and (b) for discharge power of 400 W. Hydrogen pressure was 25 Pa., Figure S3: XPS survey spectra of PTFE treated in hydrogen plasma at various forward powers. Hydrogen pressure was constant at 25 Pa and treatment time was 1 s., Figure S4: XPS surface composition of PTFE treated in hydrogen plasma at various forward powers. Hydrogen pressure was 25 Pa and treatment time was 1 s., Figure S5: ToF-SIMS spectra of the untreated PTFE: (a) positive ion spectra and (b) negative ion spectra. Figure S6: ToF-SIMS spectra of the PTFE treated in H_2_ plasma at the power of 150 W: (a) positive ion spectra and (b) negative ion spectra. The pressure was 25 Pa and treatment time was 1 s., Figure S7: ToF-SIMS spectra of the PTFE treated in H_2_ plasma at the power of 400 W: (a) positive ion spectra and (b) negative ion spectra. The pressure was 25 Pa and treatment time was 1 s., Figure S8: ToF-SIMS spectra of the PTFE treated in H_2_ plasma at the power of 800 W: (a) positive ion spectra and (b) negative ion spectra. The pressure was 25 Pa and treatment time was 1 s.
