# Supplementary figures and images for: Defluorination of Polytetrafluoroethylene Surface by Hydrogen Plasma
Source: Polymers (Basel). 2020 Nov 29;12(12):2855. doi: 10.3390/polym12122855 (PMC7760809; doi:10.3390/polym12122855)

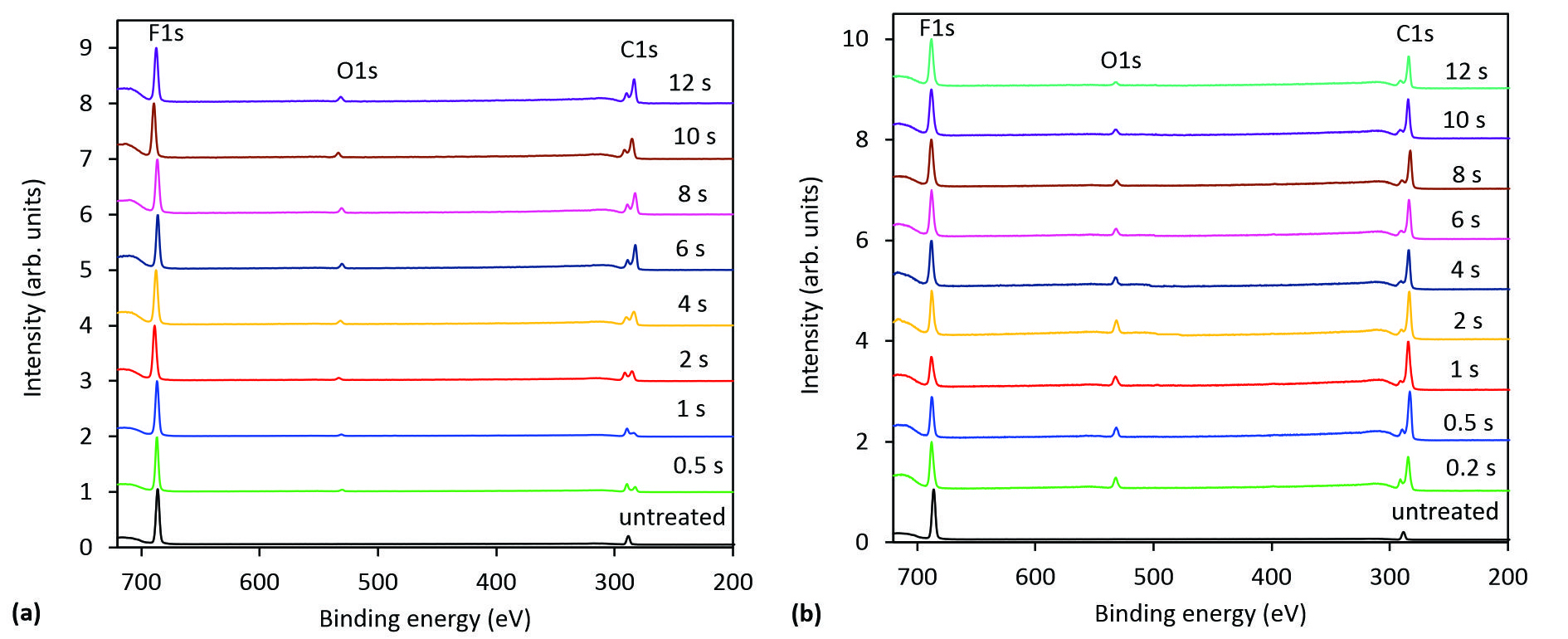

Supplement: Supplementary file 1 [file polymers-12-02855-s001.zip › Figure_S1.jpg]

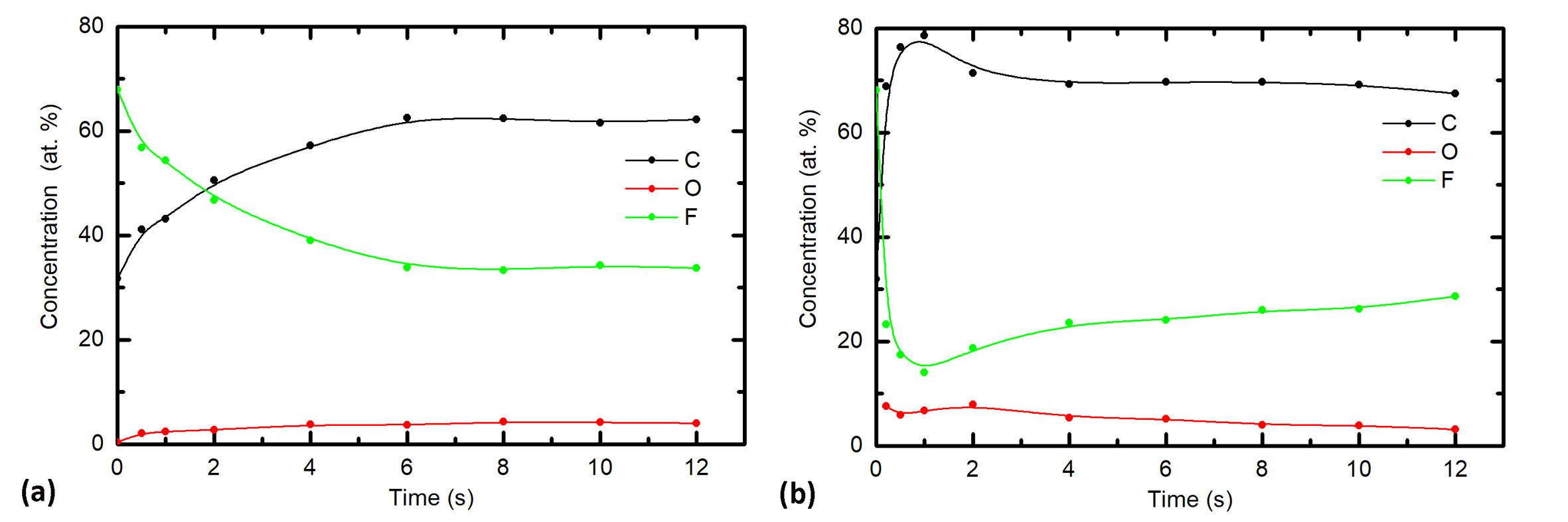

Supplement: Supplementary file 1 [file polymers-12-02855-s001.zip › Figure_S2.jpg]

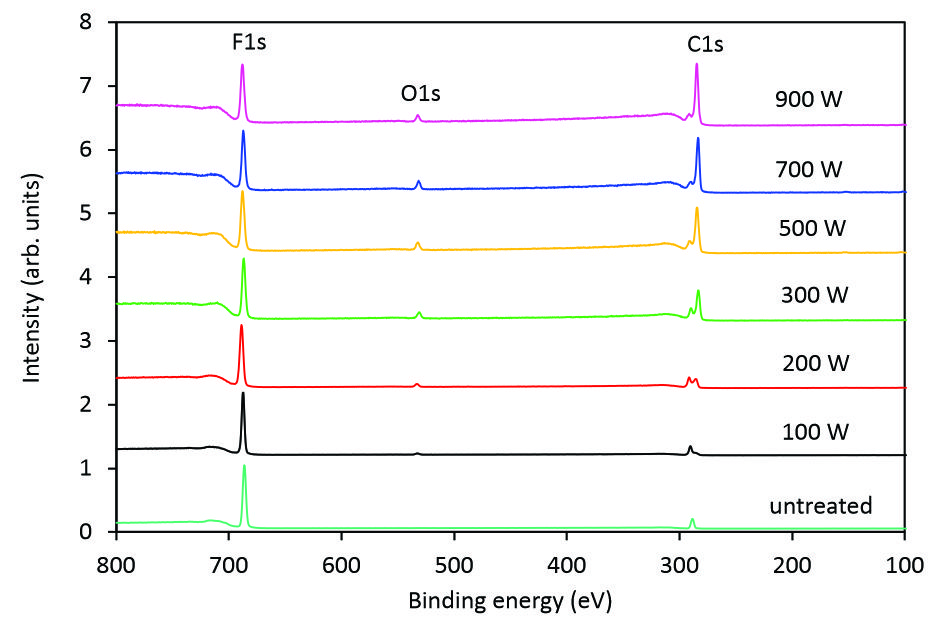

Supplement: Supplementary file 1 [file polymers-12-02855-s001.zip › Figure_S3.jpg]

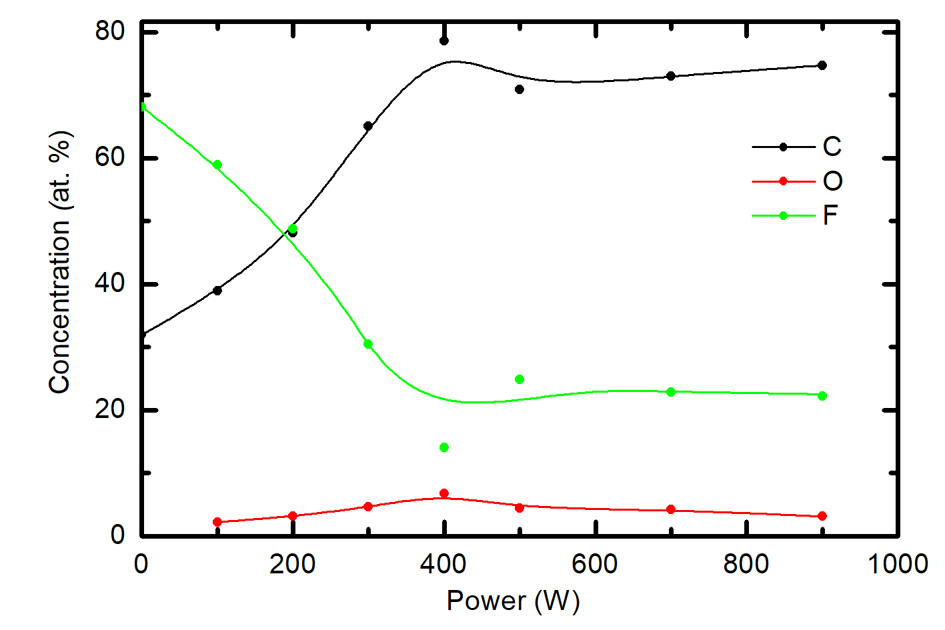

Supplement: Supplementary file 1 [file polymers-12-02855-s001.zip › Figure_S4.jpg]

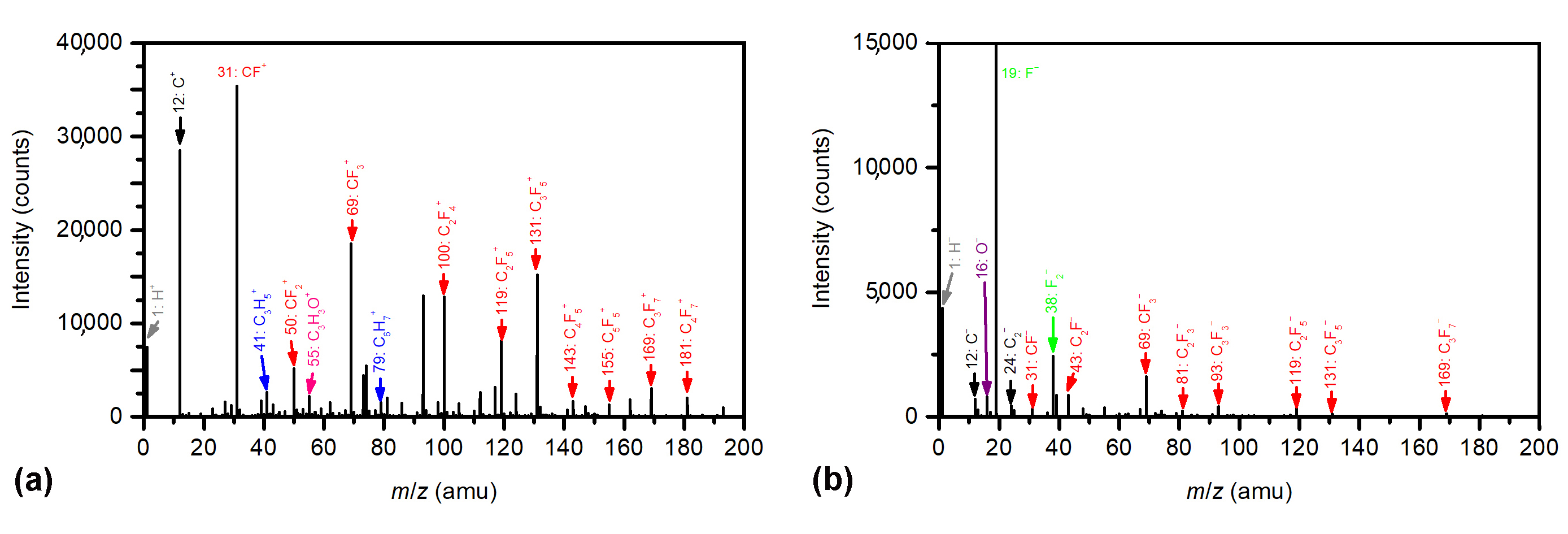

Supplement: Supplementary file 1 [file polymers-12-02855-s001.zip › Figure_S5.jpg]

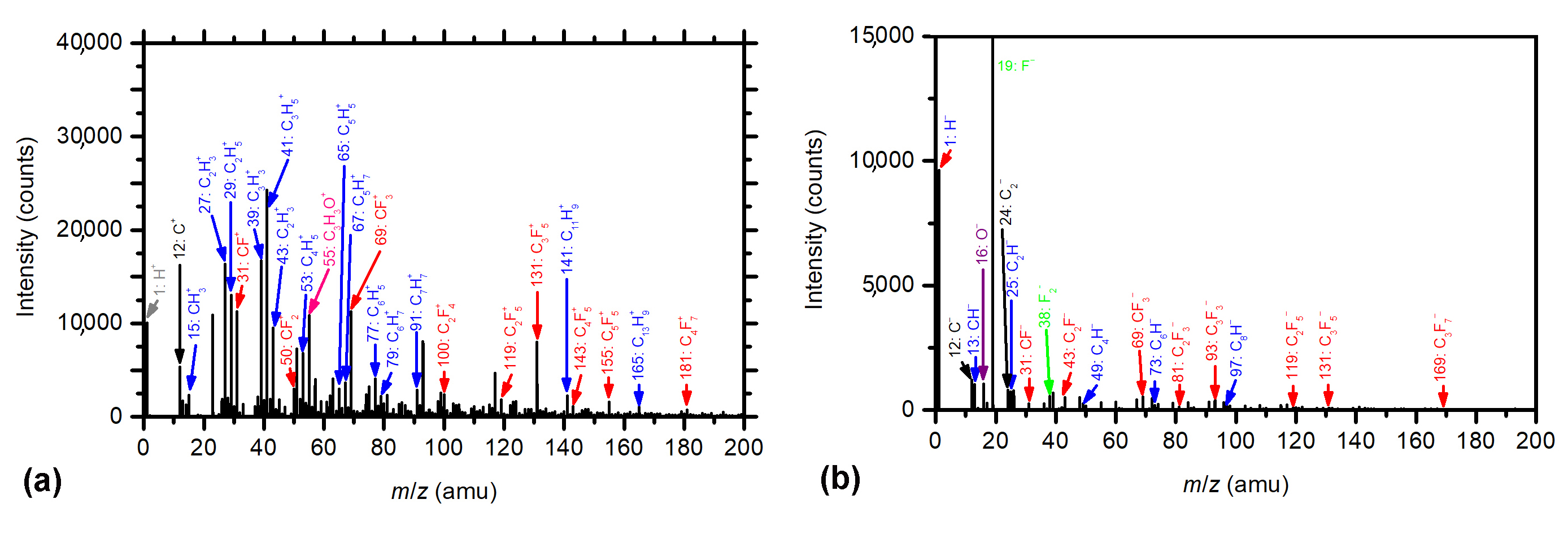

Supplement: Supplementary file 1 [file polymers-12-02855-s001.zip › Figure_S6.jpg]

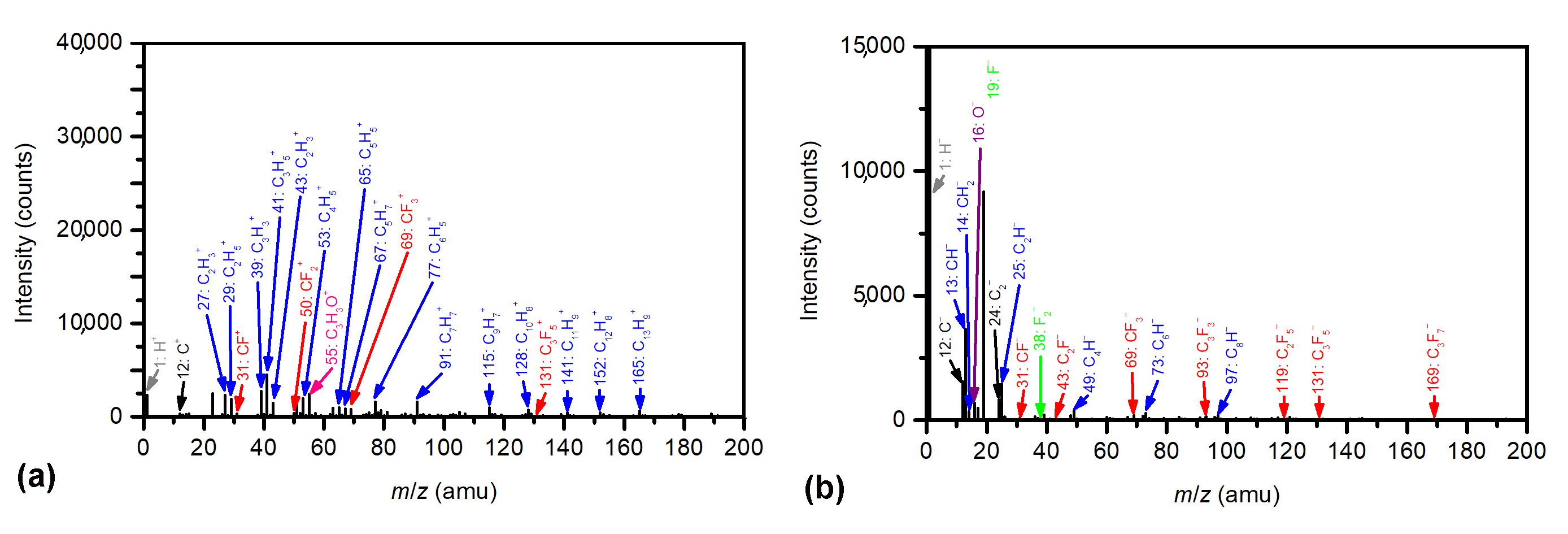

Supplement: Supplementary file 1 [file polymers-12-02855-s001.zip › Figure_S7.jpg]

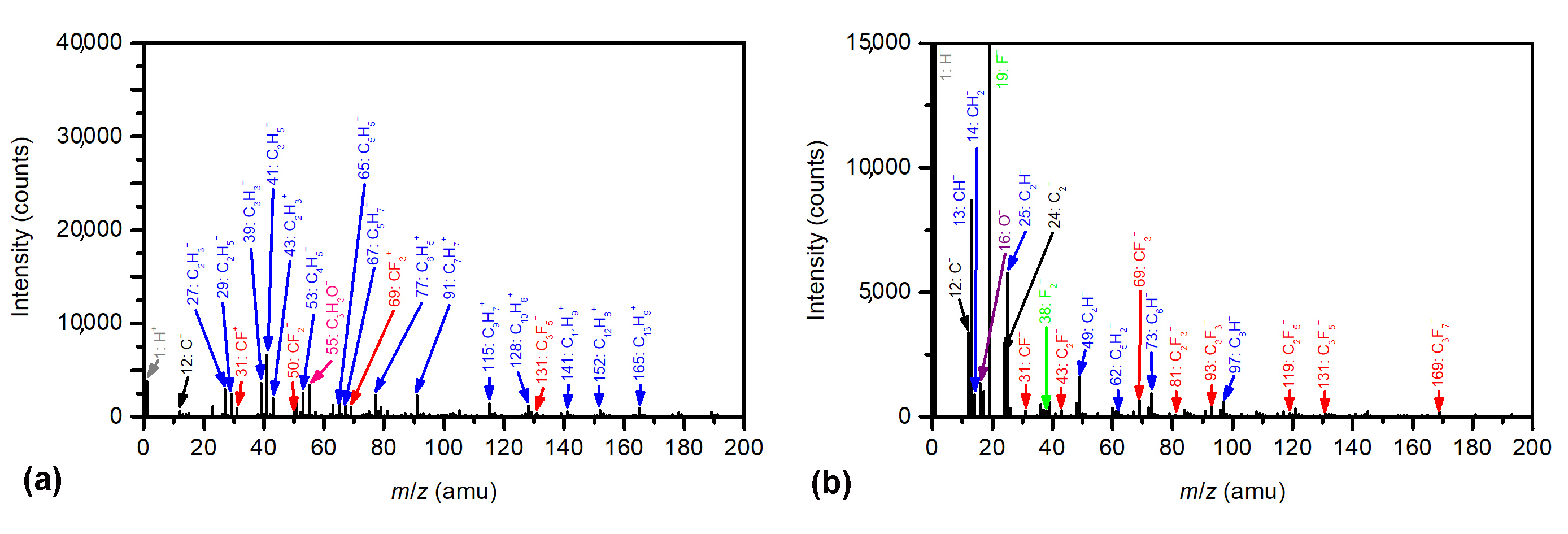

Supplement: Supplementary file 1 [file polymers-12-02855-s001.zip › Figure_S8.jpg]
